# Assessment of Ecosystem Services Provided by Macrophytes in Southern Baltic and Southern Mediterranean Coastal Lagoons

**DOI:** 10.1007/s00267-024-01955-9

**Published:** 2024-03-07

**Authors:** Esther Robbe, Linda Rogge, Jūratė Lesutienė, Martynas Bučas, Gerald Schernewski

**Affiliations:** 1 Research Unit - Coastal Seas and Society, Leibniz Institute for Baltic Sea Research Warnemünde (IOW), Rostock, Germany; 2 Marine Research Institute, Klaipeda University, Klaipeda, Lithuania; 3 International Institute Zittau, TUD Dresden University of Technology, Zittau, Germany

**Keywords:** Szczecin lagoon, Curonian lagoon, Bizerte lagoon, Scenarios, Habitats, Indicators

## Abstract

The ecological importance of macrophytes is well known and reflected in nature protection law, for example, as a key biological quality element. However, the socio-economic role, such as the impact of macrophyte presence on recreational activities, is often overlooked. The purpose of this study was to assess the human benefits (or ecosystem services) provided by macrophytes. We developed a list of 25 macrophyte ecosystem services and 79 assessment indicators based on expert knowledge and literature data. First, hypothetical scenarios of coastal lagoons were developed to assess the impact of different ecological states (i.e., macrophyte coverage) and management measures (i.e., fisheries) on the ecosystem service provision. Scenario assessments were carried out by stakeholder workshops and literature search. Second, the ecosystem service potential of submerged and emergent macrophyte habitats were assessed by macrophyte experts and literature data. Results showed that cultural services are most important in terms of the overall actual provision of ecosystem services (scenario assessment) but also showing highest potential of the hypothetical ecosystem service provision (habitat assessment). Highest overall potential is shown for reeds and tall forb communities (83 out of maximum 125), followed by seagrass beds (71) and seaweed communities (61). Our ecosystem service assessment approaches (i.e., scenario and habitat-based) using socio-cultural data (i.e., stakeholders and experts-based) and biophysical data (i.e., indicators-based) can serve as supportive tools for coastal management and policy implementation visualizing the benefits of macrophytes to humans.

## Introduction

Shallow coastal areas, especially sheltered lagoons, are characterized by their highly valuable macrophyte habitats. Macrophytes are aquatic plants and macroalgae, including emergent (e.g., reed and salt meadow species) and submerged types (e.g., seagrass, charophytes and pondweed) that can be rooted or unrooted, floating or attached. They bear important ecological functions, such as nutrient retention, carbon sequestration, coastal protection or habitats for biodiversity (Duarte et al. 2013; Newton et al. 2014; Buczko et al. 2022). Thereby, they support major socio-economic activities in coastal waters, such as leisure and tourism (i.e., by counteracting eutrophication and improving bathing water quality) as well as fisheries (i.e., by providing nursery habitat) (Newton et al. 2014; Sinkeviciene et al. 2017). In the Baltic Sea, especially reeds were historically harvested and used as building material, while nowadays macrophytes play only a minor economic role as raw material (Köbbing et al. 2013; Karstens et al. 2019). In contrast, in the Mediterranean Sea, macrophytes provide sightseeing opportunities for diving tourism, fisheries and seaweed harvesting that are big economic drivers (El Mahrad et al. 2020). These examples show that macrophytes in general provide a vast range of direct or indirect benefits to humans, also referred to as ecosystem services (Millennium Ecosystem Assessment 2005; TEEB 2010).

The concept of ecosystem services (ES) emerged in the 1960 within the field of ecological economics as a response to the need for nature conservation (Costanza et al. 1998). Since then multiple assessment approaches and classifications developed. The most popular classification system in Europe is the Common International Classification of Ecosystem Services (CICES; Haines-Young & Potschin-Young 2018). CICES differentiates between three main categories: provisioning services (e.g., charophytes for fish feeding), regulating and maintenance services (e.g., reed belts as wave attenuators), and cultural services (e.g., seagrass beds for diving). Others classification systems exist, for example, including supporting services (The Millennium Ecosystem Assessment 2005) or habitat services (TEEB 2010). For measuring ES, a huge variety of assessment methods and approaches exist, summarized and classified by Harrison et al. (2018) as socio-cultural (e.g., participatory assessments), monetary (e.g., mitigation costs) and biophysical methods (e.g., modelling).

ES of coastal waters, especially lagoons, are reviewed worldwide (Newton et al. 2018). Inventories of ES exist for specific lagoons in the Mediterranean (Velasco et al. 2018) and the Baltic Sea (Inácio et al. 2018). However, studies on ES provided specifically by macrophytes in coastal waters are scarce. They focus usually on single macrophyte species (e.g., charophytes; Schneider et al. 2015) or single services, such as carbon sequestration from seagrass (Reynolds et al. 2016). Studies also assess the ES of macrophytes under certain management measures, for example, seagrass restoration (Chen et al. 2022), seaweed cultivation (Hasselström et al. 2018) or reed harvesting (Karstens et al. 2019). In the Baltic Sea, only a few studies focus on ES provided by macrophytes (Gopal 2016; Heckwolf et al. 2021). For North African countries, interdisciplinary studies on ES including social, economic and cultural aspects are few and only recently studied: while Santoro (2023) studied the ES of agroforestry systems (i.e., traditional oases) and their main threats, El Mahrad et al. (2020) analyzed the socio-ecological importance of coastal lagoons and their management. There are only few comprehensive studies assessing ES provided by macrophytes and their habitats, especially under different management scenarios and/or environmental changes and anthropogenic pressures impacting macrophyte habitats (Lindegarth et al. 2014, Janssen et al. 2021).

Central management concerns in coastal lagoons worldwide, dominated by macrophytes, comprise of combating eutrophication (Erostate et al. 2022), regulating fisheries management (Scapin et al. 2021) and coping with coastal erosion and sea level rise (Inácio et al. 2023). Due to their proximity to the coast, macrophyte habitats are highly disturbed by natural and human-induced pressures, such as eutrophication, pollution, climate change and loss of biodiversity (Kennish and Paerl 2010). Thus, they occur in very dynamic and changing environment with varying hydrodynamics, water transparency, salinity, temperature and nutrient concentrations. This causes a change in the coverage, size and species composition of macrophyte communities (Bučas et al. 2019) as well as in their provision of ES. To address these pressures, macrophytes are well reflected within European Union (EU) water and nature policies, such as in the EU Water Framework Directive (WFD) and the Habitats Directive (HD).

The WFD (Directive 2000/60/EC) is a comprehensive water protection policy of pioneering character (Carvalho et al. 2019). Its overall aim is to achieve a “good ecological status” (GES) of all EU surface waters including “transitional and coastal waters”. The ecological status is assessed based on biological quality elements (i.e., phytoplankton, macrophytes, phytobenthos, benthic invertebrate fauna and fish), supporting physicochemical (e.g., nutrient content, water transparency) and hydromorphological elements (e.g., structure of the coastal zone) (BMUV/UBA 2022). Thus, the ecological importance of macrophytes as one key biological quality element is well reflected in the WFD, also shown by their integration in the complex assessment schemes, tools (e.g., PHYBIBCO) and parameters (i.e., ecological significance, species composition and abundance, biomass, depth limits) developed under the WFD over decades. Country-specific “Programs of Measures” to improve water quality status include, for example, agricultural practices (e.g., reducing nutrient loads from fertilization), habitat restoration (e.g., shoreline planting), and sewage treatment (e.g., reducing pollutant loads) (LUNG 2021).

Despite major efforts and numerous measures, almost 50% of all transitional and coastal waters in the EU are still not in a good or high ecological state regarding the status of macrophytes (EEA 2018). In addition to complex administrative procedures, further limitations of successfully implementing measures are the lack of financial resources, trained staff (i.e., in public administration) and required experts (BMUB/UBA 2022). While assessment methodologies require time and expertise (MariLim 2019a; 2019b), their results indicate that implemented measures have only little effect (e.g., because of the slow response time of aquatic systems) or are not always sufficiently reflected by assessment results (BMUB/UBA 2016). The multiple pressures addressed by the WFD (i.e., pollutant loads, lack of habitats) require complex combinations of measures, which are often not measurable quantitatively due to long response times of ecosystems (e.g., 10–20 years in coastal waters) (BMUB/UBA 2022).

Macrophyte habitats also play an important role in the EU Biodiversity Strategy 2030, and thereby also in the associated HD (Directive 92/43/EEC), Birds Directive (Directive 79/409/EEC) and the Natura 2000 ecological network of protected areas. One of the main objectives of the strategy is to maintain and restore ecosystems, thus the provision of ES (pillar 2 from 4; EC 2021). Actions required under the strategy include the mapping and assessment of ES and their integration, e.g., into decision making. Coastal habitats, including macrophytes, were reported to have the lowest share of “good conservation status” assessments and are in need of improvement (EC 2020b).

The concept of ES became subject of political interest recognizing its potential to support implementation processes, being partially integrated into recent EU policies (Bouwma et al. 2018). Despite numerous applications of ES assessments within EU policies (e.g., within national reports), a comprehensive integration of ES approaches is lacking, which is an ongoing challenge in the development of current EU policy and environmental legislation due to high complexity and time-consuming approaches (Schleyer et al. 2015; Bouwma et al. 2018). The benefits of ES assessments in coastal and marine management and policy implementation include, for example, to serve as a decision support tool (Rees et al. 2022) or to support participatory community engagement (Burdon et al. 2022). A simplified but holistic assessment approach for coastal areas, especially the land-sea interface covered by macrophytes, is needed that allows for an easy and fast comparison of different systems and concrete management measures (e.g., improved water quality by achieving GES), also showing their benefits and tradeoffs for human society, and thereby support implementations of EU policies.

The main purpose of this study is to develop and apply a holistic socio-economic and ecological ES assessment for macrophyte habitats in shallow coastal areas. Our aims are 1) to develop a list of ES provided by macrophytes including respective assessment indicators, 2) to assess macrophyte scenarios developed to represent different ecological states according to the WFD and management measures (i.e., coastal protection) by evaluating the relative importance of macrophyte ES and their impact of scenarios perceived by different stakeholder groups, 3) to visualize the socio-economic benefits of macrophytes by assessing the ES potential of submerged and emergent habitats (based on Natura 2000), and 4) to show the general applicability of assessment approaches within coastal management (e.g., to identify tradeoffs between tourism and conservation) and policy implementation (e.g., to show benefits of habitat recovery to achieve GES) in contrasting systems (i.e., from the Baltic Sea to the Mediterranean Sea).

## Methodology

### Study Sites

**Fig. 1:**
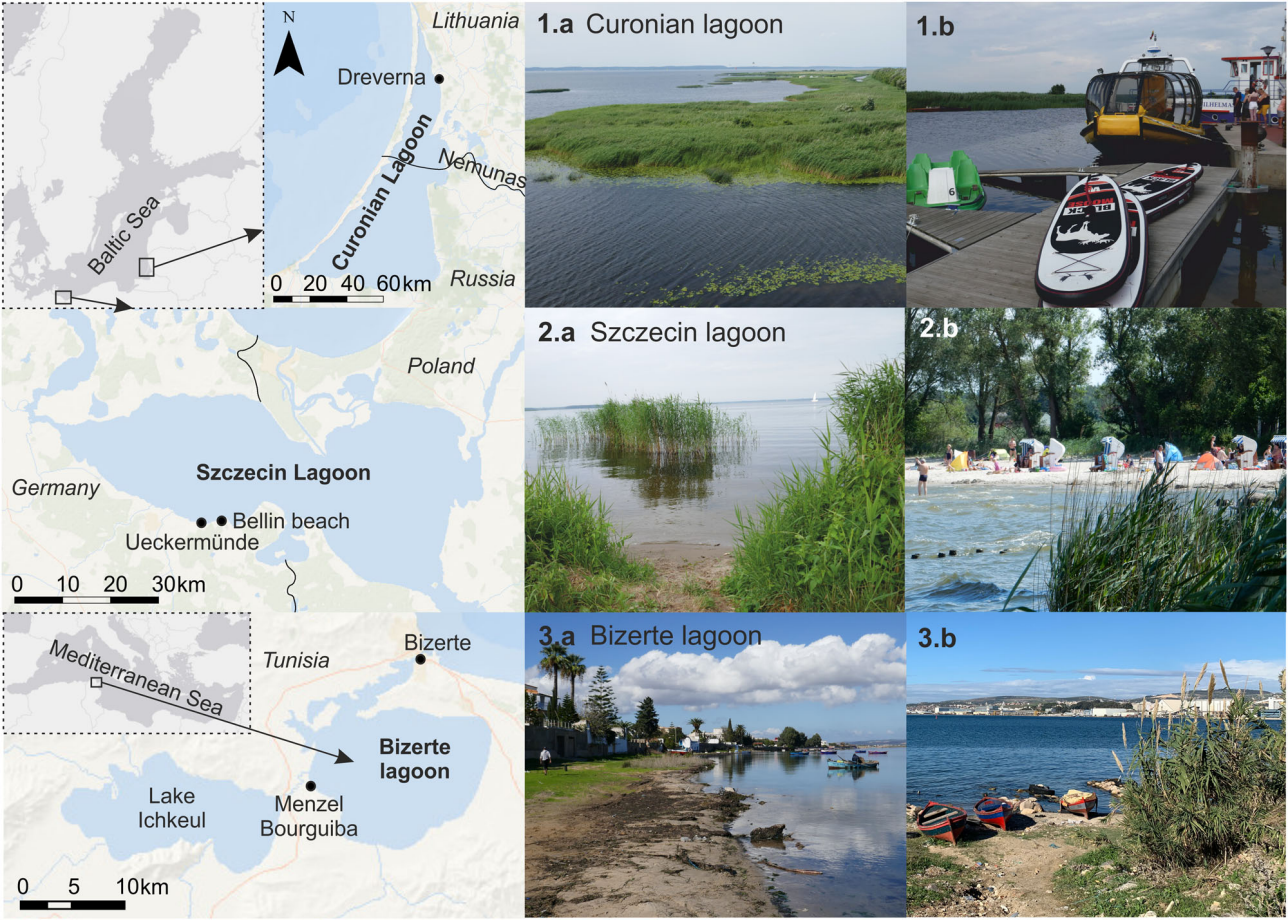
Location of the three study sites Curonian lagoon (Baltic Sea), Szczecin lagoon (Baltic Sea) and Bizerte lagoon (Mediterranean Sea). Pictures show dominating macrophyte habitats (**a**) and human uses (**b**): protected reed belts (1.a) and water sport center (1.b) in Dreverna (Lithuania), reed belts (2a) and beach tourism (2.b) in Ueckermünde (Germany), and a city beach (3.a) in Menzel Bourguiba and fishing boats (3.b) in Bizerte (Tunisia)

Our main study area are shallow coastal areas in the Baltic Sea (Szczecin and Curonian lagoon) and the Mediterranean Sea (Bizerte lagoon) with focus on coastal lagoons that are often characterized by macrophyte habitats (Fig. 1). The primary management issue of coastal lagoons worldwide is eutrophication (Erostate et al. 2022), accompanied by fisheries management (Scapin et al. 2022) and coastal erosion (Inácio et al. 2023). In order to test the general applicability of our approaches internationally, we chose and tested three lagoons (all subject to mentioned management issues) from diverse and contrasting systems (see Table 1) in terms of climate zones (i.e., warm summer climate in the Baltic, and Mediterranean climate), different physico-chemical conditions (low to high salinity; low to high turbidity; good to poor ecological states), socio-economic parameters (uses, pressures, pollution), and data availability (poor, good).

**Table 1:** Physico-chemical parameters of the three study sites: Szczecin lagoon, Curonian lagoon and Bizerte lagoon

Parameter	Szczecin lagoon	Curonian lagoon	Bizerte lagoon
Sea	SW Baltic	SE Baltic	SW Mediterranean
Country	Germany/ Poland	Lithuania/ Russia	Tunisia
Climate zone (acc. to Koeppen)	Humid continental climate (Dfb)	Humid continental climate (Dfb)	Hot-summer Mediterranean climate (Csa)
Ice coverage (days a^−1^)	59	110 (min: 12; max: 169)	0
Area (km^2^)	669 (GER: 277)	1.584 (LT: 413)	150
Largest inflowing rivers	Oder	Nemunas	Ichkeul Lake
Catchment area (km^2^)	118,000	98,200	ca. 2500
Mean depth (m)	3.8	3.8	7
Maximum depth (m)	12	14.5	12
Secchi depth (m)	< 1	< 1	< 2.5
Salinity (max/min in psu)	1–3	0.1–7	20–40
Water temperature (°C)	10 [0–20]	10 [0–21]	17 [10–29]
Trophic state	eutrophic	eutrophic	eutrophic

Data from: Schiewer (2008), Alves Martins et al. (2015), Friedland et al. (2019), Mensi et al. (2020), Stragauskaite et al. (2021)

**Fig. 2:**
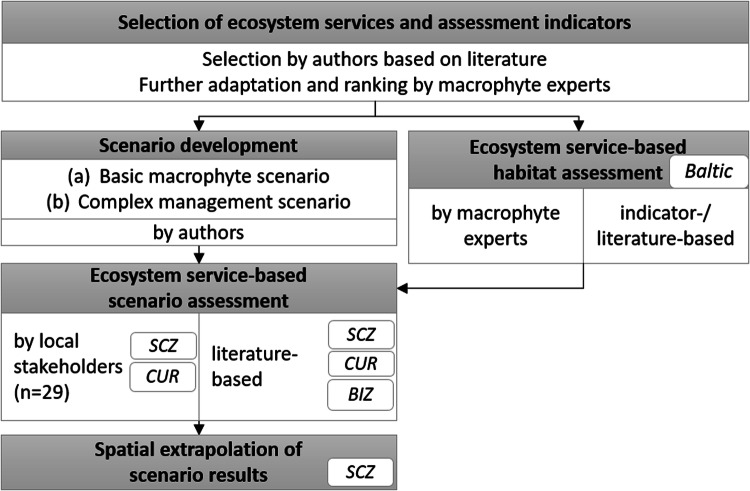
Work flow diagram of applied assessment methods and data sources (i.e., experts, stakeholders, literature) indicating applied study sites in the small boxes (SCZ Szczecin lagoon, CUR Curonian lagoon, BIZ Bizerte lagoon, Baltic - Shallow coastal areas of the Baltic Sea)

### Curonian Lagoon

The main economic driver of the biggest Baltic lagoon is the historically developed artisanal and small-scale fisheries. Anthropogenic pressures include the increased nutrient discharge from the catchment, overfishing and recreational activities along the shoreline. Despite significant efforts in preventing eutrophication, the lagoon is regarded as being in a poor ecological status (Vaičiūtė et al. 2021). Bottom sediments consist mostly of fine sand, while coarse silt and fine silty mud are common at the depth over 3 m (Stragauskaite et al. 2021). In the estuarine part of the Curonian lagoon, typical brackish water species (*Chara baltica* and *Tolypella nidifica*) are restricted to the areas affected by brackish water up to a salinity of 0.4 psu (Bučas et al. 2019). Freshwater species (*Nitellopsis obtusa*) are mainly found from the Nemunas Delta to Dreverna. *Chara contraria* and *Chara aspera* are dominant species and wide spread in the eastern littoral of the lagoon down to 2.5 m depth. Apart from abundant *Phragmites australi*s belts, the most dominant angiosperms are *Potamogeton perfoliatus, Potamogeton rutilus*, and *Stuckenia pectinat* (Stragauskaite et al. 2021). Despite a decline of submerged macrophytes due to eutrophication in 1960–1980, recent data of Sinkevičienė et al. (2017) state a current increase of charophytes. The Lithuanian part of the lagoon is designated as Natura 2000 site, under both Habitats and Birds Directives and is adjacent to the Curonian spit, which is a UNESCO world heritage site.

### Szczecin Lagoon

For centuries, the main economic sector of the lagoon has been fisheries and, more recently, also tourism. Thus, many settlements nearby directly or indirectly rely on the goods supplied by the lagoon. Nutrient enrichment of the lagoon causes an unsatisfactory ecological state, classified as ‘poor’ according to the WFD (Friedland et al. 2019). Riverine nutrient loads from agriculture and urban areas keep the lagoon in a polytrophic to hypertrophic state. Most dominant habitats include common reed (*Phragmites australis*), usually occurring in bands or patches, and charophytes (*Chara spp.)*. Despite the recent increase in macrophytes, historical records for the Szczecin lagoon show a strong decline in macrophyte habitats, mainly due to eutrophication, from an estimated 36% coverage 130 years ago to a current state of 12% coverage (Schernewski et al. 2023). The lagoon is a designated Natura 2000 site with additional huge parts under landscape protection, two adjacent national parks in the coastal area and several nature reserves.

### Bizerte Lagoon

Since the 1950s, the lagoon has been being highly modified and intensively used by humans, mainly for fishing activities, and mussel and oyster farming (Khammassi et al. 2019). The lagoon is a highly industrialized area with around 130 industrial factories located nearby (El Mahrad et al. 2020). Thus, the main pressures include discharges from urban and industrial pollution sources (i.e., textile dyeing industry and metallurgic factory, domestic sewage) (Zaabar et al. 2017). Only 15 years ago, the ecological state was considered to be in an overall satisfactory condition (Afli et al. 2008). However, due to the high nutrient inputs a current change towards eutrophication is observed (Zaabar et al. 2017). Dominant sediments are muddy sands covered by main macrophyte compositions of seaweeds (*Ulva lactuca*, *Cladophora sp*., *Gracilariopsis longissima* and *Gracilaria bursa*-*pastoris*) and seagrass (*Cymodocea nodosa*) (Zaabar et al. 2017). Compared to other Mediterranean waters, Bizerte lagoon has a low species richness and abundance due to its extremely fluctuating environmental conditions. Consequently, a major ecological constraint is the high seasonal variability in temperature, salinity, and nutrient concentration (Zaabar et al. 2017). The lagoon is connected to the Ichkeul lake which is a National Park and UNESCO world heritage site.

### Ecosystem Service Assessment

Our ecosystem service assessment consists of five components (Fig. 2). Based on the selection of ecosystem services and assessment indicators, we followed two main assessment approaches. First, after scenario development, we conducted ecosystem service-based scenario assessments (i.e., stakeholder and literature-based) to evaluate the perceived impacts of different ecological states of lagoons on service provision by macrophytes (including spatial extrapolation). Second, we applied an ecosystem service-based habitat assessment (i.e., expert and literature-based) to compare the service potential of different macrophyte habitats typical for coastal waters of the Baltic Sea, visualizing their socio-economic benefits. These results were also combined with scenario assessment results.

### Selection of Ecosystem Services and Assessment Indicators

With the aim to develop a list of macrophyte ecosystem services and respective assessment indicators, we provide the basis for the overall objective of developing a generally valid ES assessment scheme for macrophyte habitats in shallow coastal areas. Following selection criteria were applied: socio-economic and ecological relevance, frequency, tolerable assessment time for stakeholders/ experts, and balance between ES categories. The set of ecosystem services were derived from the ecological functions and processes related to macrophytes (Hossain et al. 2017), then defined and classified according to CICES V.5.1 based on Haines-Young and Potschin-Young (2018) and Burkhard et al. (2014), adapted according to Gopal (2016) and von Thenen et al. (2020). We then compiled a set of assessment indicators for each service. The indicators are based on literature (von Thenen et al. 2020), but also complemented and adapted with the help of local macrophyte experts in separate, individual and remote assessments. They were asked to rank the three most suitable and important indicators per service by a ranking score of 1 to 3 (most suitable). The selection list of indicators comprises the three highest-ranked indicators (according to the sum of all expert scores). Afterwards, we pre-tested the importance and comprehensibility of the services (including descriptions) in an expert workshop (Workshop 1 in Table 2), and further adapted and tested them again with experts from local study areas.

**Table 2:** List of stakeholder workshops in which the ecosystem service-based scenario assessment approach was applied

	Date	Location	Study site	Scenarios	# Participants	Stakeholder groups
1	03.09.20	Rostock, Germany	Szczecin lagoon	Complex management scenarios (0.b, 1.b)	12 (German)	Academia & science: local experts (100%)
2	07.08.22/ 18.08.22	Rostock, Germany	Szczecin lagoon	Basic macrophyte scenarios (0.a, 1.a)	12 (German: 25%, Dutch: 75%)	Academia & science: local experts (42%), public authorities (50%), public audience (8%)
3	03.10.22	Klaipeda, Lithuania	Curonian lagoon	Basic macrophyte scenarios (0.a, 1.a)	5 (Lithuanian)	Academia & science: local graduate students (100%)

### Scenario Development

**Fig. 3:**
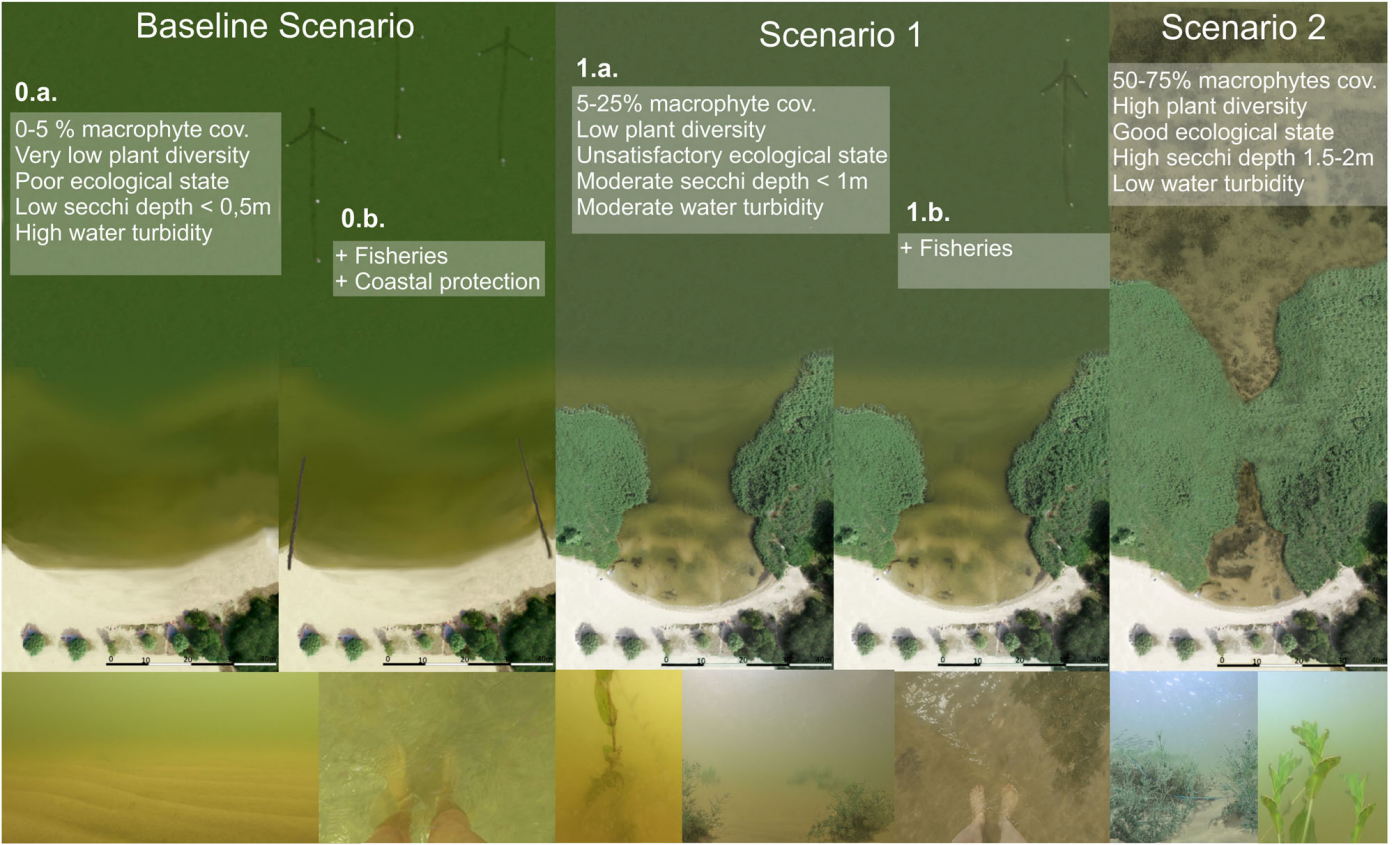
Visualization of basic macrophyte scenarios used for stakeholder and literature-based assessments by orthophoto view of coastal area transect (above) and its underwater view (below): The Baseline Scenario (0.a.) represents a poor ecological state, Scenario 1.a. a moderate ecological state and Scenario 2 a good ecological state (GES). Additional scenarios presented for assessment include complex management context (fisheries: high intensity (0.b.) and sustainable yields (1.b); and coastal protection: installed groins (0.b.), protection function by macrophyte stand (1.b.)

In the scenario building, we developed scenarios that aim to assess the impact of a prospective good ecological state (GES) of coastal lagoons (basic macrophyte vs. turbid water scenarios) and of certain management measures (coastal protection and fishery) on ecosystem service provision as perceived by stakeholders. For this, we developed five hypothetical scenarios showing a coastal transect typical for the Szczecin and/or Curonian lagoon based on the current state of Bellin beach (Szczecin lagoon, Fig. 1) which was taken as references state (1.a) (Fig. 3). In the Curonian lagoon, similar conditions can be found, for example, in Dreverna (Fig. 1) assuming the transect to be representative for both lagoons (also confirmed by local experts). We differentiate between basic macrophyte scenarios (0.a., 1.a. and 2) representing different ecological states according to the WFD (poor, moderate, good) and complex management scenarios (0.b. and 1.b.) depicting concrete management measures, i.e., regulating fisheries and coastal protection structures. Hereby, we aim to identify possible tradeoffs or synergies between achieving a GES (acc. to the WFD), coastal protection measures and different fishing intensities with regard to the recovery of macrophytes. These scenarios are used for stakeholder-based workshops and the application of ecosystem service-based scenario assessments.

- *Baseline scenario 0* (poor ecological state) represents a heavily eutrophic water body with almost entire macrophyte disappearance; in addition, the complex management scenario (0.b.) includes concrete management demands as coastal protection measures (wooden groins) and a high fishing intensity (three fish traps).
- *Scenario 1* (moderate ecological state) shows a narrow reed belt along the shore, representing the most common current state of the study sites; in a complex management scenario (1.b.) macrophyte belt performs coastal protection function, therefore artificial protection infrastructure is no more needed, whereas a low fishing intensity (one fish trap) was added – corresponding to achieved sustainable fishery landings.
- *Scenario 2* (good ecological state) represents a state after possible conservation or nature protection measures are implemented without any commercial fisheries, and no need for artificial coastal protection; the system shifts from domination of phytoplankton production to domination of macrophyte production and increase in habitat coverage (no need for complex management scenario).

### Ecosystem Service-based Scenario Assessment

The scenario assessment was further performed based on ecosystem service assessment approach carried out by three participatory workshops with different stakeholder groups (see Table 2). In Workshop 1 (Szczecin lagoon) targeting the local scientific community, 12 local environmental researchers (or coastal management experts) from five different research institutes and from relevant disciplines (ecology, biology, geography) participated and assessed the complex management scenarios (0.b, 1.b). In Workshop 2 and 3 the basic macrophyte scenarios (0.a, 1.a) were assessed. For assessing the Szczecin lagoon, Workshop 2 aiming at practitioners and coastal managers comprised of 12 stakeholders from different sectors (science, government and NGOs), who worked in coastal waters and macrophyte management (legislation, conservation, monitoring). Workshop 3 performed in Lithuania targets local graduate students in the related fields (marine biology, ecology, coastal engineering) to assess scenarios in the Curonian lagoon ecosystem.

Workshops were conducted in groups, face-to-face or in person, online or combined. The duration of the workshops was between 90 and 120 min, including introduction (~30 min), assessment (~30 min) and discussion (30–60 min). In the introduction, the scenarios, their visualizations and the assessment method were presented. First, we asked them to indicate the “Relative Importance (RI)”, which indicates how important each ES is perceived relatively to the overall ES provision of the given transect. By doing this, we evaluated the suitability of selected ES. The scoring scheme ranges from “not important” [0], “low” [1], “moderate” [2], “high” [4] to “very high importance” [8] based on Robbe et al. (2021). Second, experts assessed the relative “Change” of Scenarios 1 and 2 compared to the Baseline scenario. These values based on stakeholders´ perceptions and knowledge indicate how the ES are changed or impacted by different states of the given transect. The scoring scheme ranges from high [+/−3], moderate [+/−2], low [+/−1] negative or positive change or no change [0]. In Workshop 1, the additional score of [+/−4] was tested. Stakeholders indicated their level of expertise (1 = low, 2 = moderate, 3 = high) for ES, macrophyte ecology, and management and policy. As a basis for discussion, participants were asked to explain their extreme low or extreme high values in order to identify under-/overestimations, misunderstandings and/or misconceptions. In particular, services with high standard deviations (SD > 1.5) of the relative importance values and the impact scores indicate a need for further discussion and clarification. To calculate the SD of the logarithmic scale of RI (0, 1, 2, 4, 8), we converted RI values into an arithmetic scale, assuming a normal distribution. In Workshop 1, experts were allowed to change values after discussion (in case of misunderstandings). For a quick data validation analysis of Workshop 1 (experts), 50% of the experts (three with each high and low expertise) were asked to repeat the assessment after one week.

Complementary to the stakeholder-based assessments, separate literature-based assessments compiled area-specific and local literature data for each ecosystem service using scientific databases (i.e., Web of Science, Google Scholar). When not available from the study sites, data from similar areas in the Baltic or Mediterranean Sea were used. Following a semi-structured narrative approach, most recent to 30-year-old literature was scanned for keywords of each ecosystem service, their descriptors, indicators and respective lagoon. Exemplary search strings were as following: (“Szczecin lagoon”) AND (“reed”) AND (“coastal protection” OR “erosion rate” OR “height of vegetation”). Based on this compiled knowledge, we followed the same approach as the stakeholders described above, allocating the RI and impact scores according to literature found. With this method we can compare the perception of stakeholders with the scientific view reflected in scientific literature. Using literature survey approach, we analyzed the basic macrophyte scenarios (0.a., 1.a. and Scenario 2) in the Mediterranean Sea and in the Bizerte lagoon. However, due to the still ongoing and projected eutrophication processes, the Baseline scenario for the Bizerte lagoon represents the predicted eutrophic state in the future (poor ecological state), which is compared to the current good ecological state (Scenario 2) and to a moderate ecological state (Scenario 1).

### Spatial Extrapolation of Scenario Assessment

In the next step, we carried out a spatial extrapolation exemplarily for the scenario assessment results of the Kleines Haff of the Szczecin lagoon (German part) to test the applicability of our results on water body level. For transferring our small-scale results to large-scale system level, we used the most robust data of the three lagoons (Szczecin, Curonian and Bizerte lagoon) being provided for the real scenario transect of Bellin beach (Szczecin lagoon). Thereby, we aim to identify areas for which our scenario results are relevant and applicable, and to identify areas most suitable for management and policy measures to mitigate tradeoffs between human use and nature protection. We combined spatially explicit data on human uses (i.e., urban settlements, protected areas, recreational use) and habitat distribution (i.e., submerged and emergent habitats based on depths) with our scenario assessment results (i.e., RI and impact values).

For this, we first mapped the current submerged vegetation (i.e., angiosperms and charophytes) adopted from Paysen (2016) and Porsche et al. (2008) (Fig. 3). Based on the assumptions that submerged vegetation expands up to a depth limit of 3 m according to Porsche et al. (2008), we mapped the potential submerged distribution of macrophytes. We defined a coastal zone of 1000 m along the shoreline. We then chose areas for extrapolation that show similar conditions as given in the transect of Scenario 1 (current state of Bellin beach) in terms of vegetation, beach access and proximity to urban settlements, and thus recreational use (i.e., beach tourism). By linking spatial data and the RI values of scenario assessment, we mapped the current spatial use for the extrapolated areas, including macrophyte habitats, recreational use (services C1 and C2) and fisheries (service P2), representing Scenario 1. For Scenario 2, we mapped the potential spatial use under the assumption of achieving the GES (i.e., increased water transparency and habitat expansion), establishing nature-protected areas and subsequently banning fisheries. We evaluated the increase or decrease in spatial uses (i.e., habitats, recreational use, fisheries, nature protection) of Scenario 2 (potential) compared to Scenario 1 (current) and linked these to our scenario assessment results (i.e., impact values).

### Ecosystem Service Provision Potential by Macrophyte Habitats

**Fig. 4:**
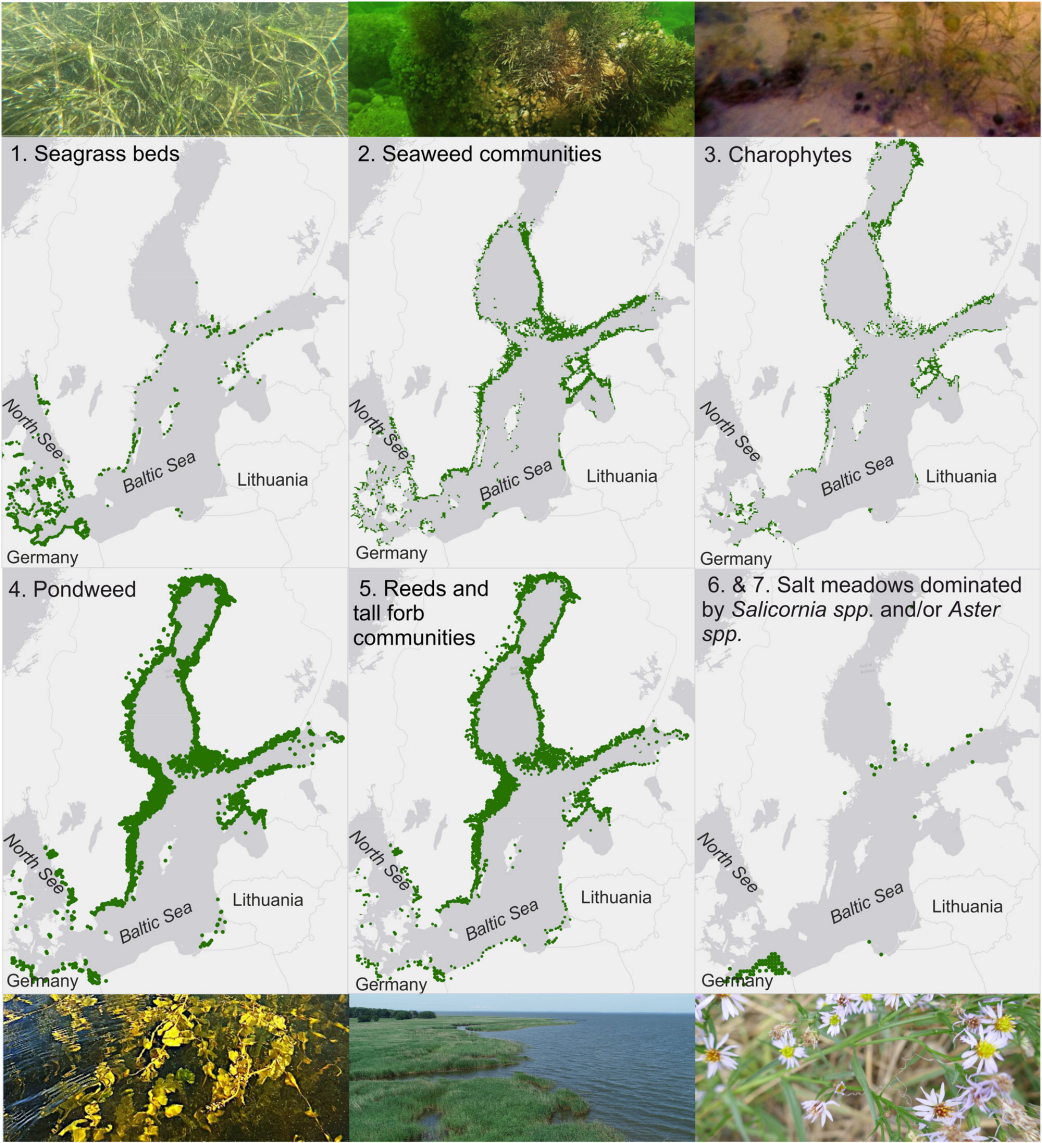
Macrophyte habitats (according to EUNIS and HD classifications) and their current distribution in the Baltic Sea that were used by macrophyte experts for the habitat assessment. Observational data for distribution maps were received from HELCOM (2023) and GBIF (2023)

We aim to assess and compare the ES potential of submerged and emergent macrophyte habitats using expert knowledge and indicators. Our assessment units constitute for ideal and hypothetical scenarios based on EUNIS and HD classifications (detailed descriptions in Online Resource 1) and show seven different macrophyte habitats and species along the land-sea gradient (see Fig. 4): 1) seagrass beds, 2) seaweed communities, 3) charophytes, 4) pondweed, 5) reeds and tall forb communities, 6) salt meadows dominated by *Salicornia*, and 7) salt meadows dominated by *Aster tripolium*. We selected the macrophyte habitats according to following criteria: a. most dominant species and habitats of the study areas (i.e., lagoons and shallow coastal areas), and b. most important species and habitats from a management perspective (i.e., local iconic species, cultural and economic value). We assume a total area of 100 m^2^ for all habitats, which is the minimum area to be considered as such by the HD. They are based on and adapted from the definitions of the HD and the categories from the European Nature Information System (EUNIS 2022). Detailed descriptions of the habitats can be found in Online Resource 1. We consider all shallow coastal areas of the Baltic Sea between 1.5 and 12 psu.

Due to the high complexity and in-depth knowledge required, the habitat assessment approach involved only macrophyte experts. In total, eleven experts from two countries (Germany: 82%, Lithuania: 18%) carried out the assessment individually and remotely between June and September 2022. Experts were provided with an assessment guideline including detailed habitat descriptions (Online Resource 1) and distribution maps based on observational data from HELCOM (2023) and GBIF (2023) (Fig. 4). Main criteria for expert selection was their field of expertise and work duration in this field (minimum of 5 years) and in the region (Baltic Sea), i.e., for each macrophyte habitat at least one expert specifically working on one single habitat was selected. Ten experts hold doctoral degrees related to macrophyte ecology (i.e., aquatic, benthic, landscape, coastal ecology, marine biology) including two professorships coming from five different research institutions, while one expert came from a state authority being responsible for coastal and marine water quality control. For analysis and interpretation of results, experts indicated their level of expertise for ES, management and policy, and each macrophyte habitat (detailed list in Online Resource 4). First, experts assessed the “Ecologically Sustainable ES Potential” for each habitat and service. In Balzan et al. (2018), ES potential is referred to as the capacity of an ecosystem to provide an ES. Burkhard et al. (2012) define ES potential as ‘the hypothetical yield of selected ecosystem services’. Here, we further define the ES potential as the “hypothetical yield” provided by the ecosystem without disturbing the natural ecosystem state nor causing a regime shift. The scoring ranges from “very low” [1], “low” [2], “moderate” [3], “high” [4] to “very high” [5] potential or “none” [0]. They had the opportunity to comment on their values, ask questions and/or express uncertainties in an extra column. Experts spent on average 60 to 90 min for the whole assessment, including the ES assessment and indicators selection. We carried out a correlation analysis for the level of expertise and the services using the R package ‘CORRPLOT’ (Wei and Simko 2021).

In a second step, we carried out a literature-based assessment applying the developed indicator list (see Table 3) and using the same habitats and scoring scheme as the experts. For the literature-based assessment, we used automated literature search scanning the database Web of Science (WoS). Literature was searched for quantitative data on the selected indicators. We aimed to find comparable data at least for one indicator from the developed list per ecosystem service and complemented this by an additional indicator “Number of WoS articles” indicating the scientific relevance of given keywords, which we assume represents the ES potential. The general search string includes the regional focus, the macrophyte habitats and the ecosystem service descriptors as following: (“Baltic”) AND (“seagrass” OR “zostera”) AND (“bioethanol” OR “bioenergy” OR “ethanol production”) (see Online Resource 5 for full list of search strings). The highest value found in literature was designated a score of 5, while the others were automatically classified according to the following scheme, as also used in the Marine Ecosystem Services Assessment Tool by Inácio et al. (2018): 5 (1 to 1/1.3), 4 (1/1.3 to 1.7), 3 (1/1.7 to 1/2.5), 2 (1/2.5 to 1/4.1) and 1 (1/4.1 and less).

**Table 3:** List of selected ecosystem services (P – provisioning, RM – regulating and maintenance, C – cultural) provided by macrophytes and assessment indicators ranked by experts (see Online Resource 3 for full list including units and sources)

Ecosystem services	Description	Indicators
P1: Marine plants used for human nutrition	Use of wild and cultivated plants as human food source or supplements, e.g. seaweeds or reed sprouts for consumption	1) Amount of harvested biomass, 2) Total sales or market value of harvested biomass, 2) Abundance/ biomass of (potential) stock/habitat, 3) Generated income or employment (farmers, processors and/or vendors), and other: nutritional value of target species (e.g. vitamins or antioxidative capacity)
P2: Marine plants used as material (direct use, processing)	Use of wild and cultivated plants incl. fibers as material, e.g. as fertilizer in agriculture or reed for thatched roofs	1) Amount of harvested biomass, 2) Abundance/ biomass of (potential) stock/habitat/raw material, 3) Abundance/ number of species with potential/actual use for processing
P3: Marine plants used for energy	Use of wild and cultivated plants as biomass for energy conversion	1) Amount of energy produced by harvested biomass, 2) Amount of harvested biomass, 3) Abundance/ number of species with potential/actual energetic value, 3) Area or coverage of potential stock/habitat, and other: Biochemical methane potential (BMP)
P4: Marine animals used for nutrition, material or energy	Wild and reared animals, e.g. fish and mussels used as source for human nutrition, direct use, processing or for energy conversion	1) Amount of harvested biomass/catch/landing, 2) Abundance/ biomass of (potential) stock/habitat, 3) Total sales or market value of products
P5: Genetic material of marine plants	Seeds and spores and other plant materials that can be used to maintain or establish a new population (seed collection) or develop new varieties	1) Number of species/genes utilized, 2) Abundance/ number of species with potential/actual useful genetic material, 3) Quality of species with potential/actual useful genetic material
P6: Genetic material of marine animals	Marine animals (e.g. fish or mussels) used for replenishing stocks or breeding of new species, e.g. breeding of new oysters’ strains	1) Abundance/ number of species with potential/actual useful genetic material, 2) Number of species/genes utilized, 3) Number of patents and published articles, 3) Quality of species with potential/actual useful genetic material
RM1: Mediation of wastes and pollutants	1) Bio-remediation; 2) Filtration/ sequestration/ storage/ accumulation by micro-organisms, algae, plants, and animals	1) Nitrogen removal/ storage, 2) Phosphorus removal/ storage, 3) Coastal recreation associated with reduced nutrient concentration
RM2: Mediation of nuisances of anthropogenic origin	1) Smell reduction, e.g. shelter belts that filter particulates that carry odors; 2) Visual Screening: Shelter belts to screen unsightly things e.g. reed belts	1) Elevation/ height of vegetation, 2) Length of coastal vegetation, 3) Abundance/ biomass of coastal vegetation (density)
RM3: Mass stabilization and control of erosion rate	Sediment stabilization controlling or preventing erosion/ mass movements e.g. by seagrass meadows	1) Area or coverage by emerged, submerged or intertidal vegetation, 2) Shoreline erosion and/or accumulation rate, and other: Stem density and seasonality of plants (e.g. perennial, annual, litter production,…)
RM4: Hydrological cycle and water flow regulation	Regulating water flows and coastal protection, e.g., coastal habitats/ natural levees reducing wave energy and providing flood protection	1) Wave attenuation potential, 2) Shoreline erosion rate, 2) Replacement cost for coastal protection
RM5: Wind protection	Shielding people from wind e.g., reed belts alleviate onshore wind	1) Elevation/ height of vegetation, 2) Abundance/ biomass of coastal vegetation (density), 3) Length of coastal vegetation, and other: configuration of coastal vegetation including length and width, stem density
RM6: Lifecycle maintenance and pollination	Seed and/ or gamete dispersal for population maintenance, e.g., providing a habitat for native pollinators	1) Extent of nursery and feeding areas, 2) Species abundance, richness and distribution, 3) Juvenile fish density
RM7: Biodiversity and habitat	Maintaining nursery populations and habitats (incl. breeding grounds) for wild plants or animals, e.g., seagrass beds as nursery habitat for commercial fish stock	1) Species abundance, richness and distribution, 2) Extent of nursery and feeding areas, 3) Total number or coverage of protected areas, 3) Habitat health status (Habitat fragmentation index)
RM8: Pest and disease control	Providing a habitat for native pest (incl. invasive species) and disease control agents, e.g., microbial antagonists for the control of postharvest diseases	1) Presence and distribution of pests/ diseases, 2) Presence and distribution of pathogens, 3) Presence and distribution of alien species, and other: algae blooms, water exchange time
RM9: Nutrient regulation (soil quality)	Decomposition and fixing processes and their effect on sediment quality, e.g. sequester and store nutrients in sediment enhancing remineralization processes	1) Nitrogen removal/ storage, 2) Phosphorus removal/ storage, 3) Carbon stock
RM10: Regulation of water conditions	Controlling chemical condition of salt water by living processes, e.g., water purification by marine plants or animals	1) Oxygen concentration, 2) Primary production, 3) Nitrogen removal/ storage
RM11: Atmospheric composition and conditions	Regulation of air, temperature and humidity, including ventilation and transpiration, e.g., carbon sequestration	1) Primary production, 2) Carbon sequestration, 3) Carbon stock
C1: Recreation and tourism (active)	Using the environment for sports and recreation, and to help stay fit, e.g., swimming, water sports, fishing	1) Total number of tourists, 2) Available beach or recreational area, 3) Number of suppliers of recreational activities (boating, surfing, diving..)
C2: Recreation and tourism (observational)	Using nature to distress, e.g., watching seabirds, plants or marine mammals	1) Number of viewpoints/ birdwatching points, 2) Species abundance, richness and distribution, 3) Total income or market value of ecotourism, 3) Presence of endangered, protected, iconic and/or rare species or habitats
C3: Research and traditional knowledge	Studying nature for scientific purpose or the creation of traditional ecological knowledge	1) Presence of endangered, protected, iconic and/or rare species or habitats, 2) Number of patents and published articles, 3) Total income or value of research funds, and other: Number of local research institutes
C4: Education and training	Using nature for educational purposes, e.g., university courses, in-situ teaching or field trips	1) Number of educational activities (in-situ teaching or field trips), 2) Presence of endangered, protected, iconic and/or rare species or habitats, 3) Revenues or number of documentaries, books and other educational publications, and other: Educational capacity (beds in youth hostels, camp sites, educational stands…)
C5: Culture and heritage	Things in nature that help people identify with history or culture of where they live or come from; that contribute to cultural heritage.	1) Social perception of identity/heritage, 1) Presence of endangered, protected, iconic and/or rare species or habitats, 2) Number of cultural events related to the area
C6: Landscape aesthetics	The inherent beauty of nature	1) Number of pictures published on social media, 2) Hedonic pricing: cost of property next to aesthetic sites, Resident population/ Net migration, and other: Landscape richness
C7: Symbolic or religious meaning	Things in nature that have symbolic or spiritual meaning, or sacred and religious meaning.	1) Number of symbolic or religious sites (church, monuments..), 2) Number of religious events (ceremonies, wedding, funerals..), 3) Presence of endangered, protected, iconic and/or rare species or habitats
C8: Natural heritage and conservation	Things in nature that should be conserved and preserved for future generations, and have a non-use value (also existence, option or bequest value)	1) Total number or coverage of protected areas, 2) Willingness-to-pay to maintain/preserve/conserve, 3) Presence of endangered, protected, iconic and/or rare species or habitats

## Results

### Compilation of Ecosystem Services and Assessment Indicators

In order to provide a generally valid ES assessment scheme for macrophyte habitats, we developed a list of services and indicators. As a result of our literature review and expert consultations, we found 25 services relevant for assessing macrophyte habitats (Table 3). For each service, we listed 3 to 14 relevant indicators suggested by the literature review. Experts were asked to select the three most suitable indicators and score their suitability by giving a rank from 3 (most suitable) to 1. The full list shows the individual rankings and values of each participating expert (see Online Resource 2). Based on the opinion of 11 macrophyte experts, we chose 79 indicators out of a total of 174 indicators pre-selected from literature by their total sum of expert rankings (including four indicators of equal sum). Ten additional indicators were mentioned and ranked by experts. This comprehensive list of services and indicators served as the basis for our further literature surveys and assessments.

### Evaluation of the Relative Importance (RI) of Ecosystem Services

**Fig. 5:**
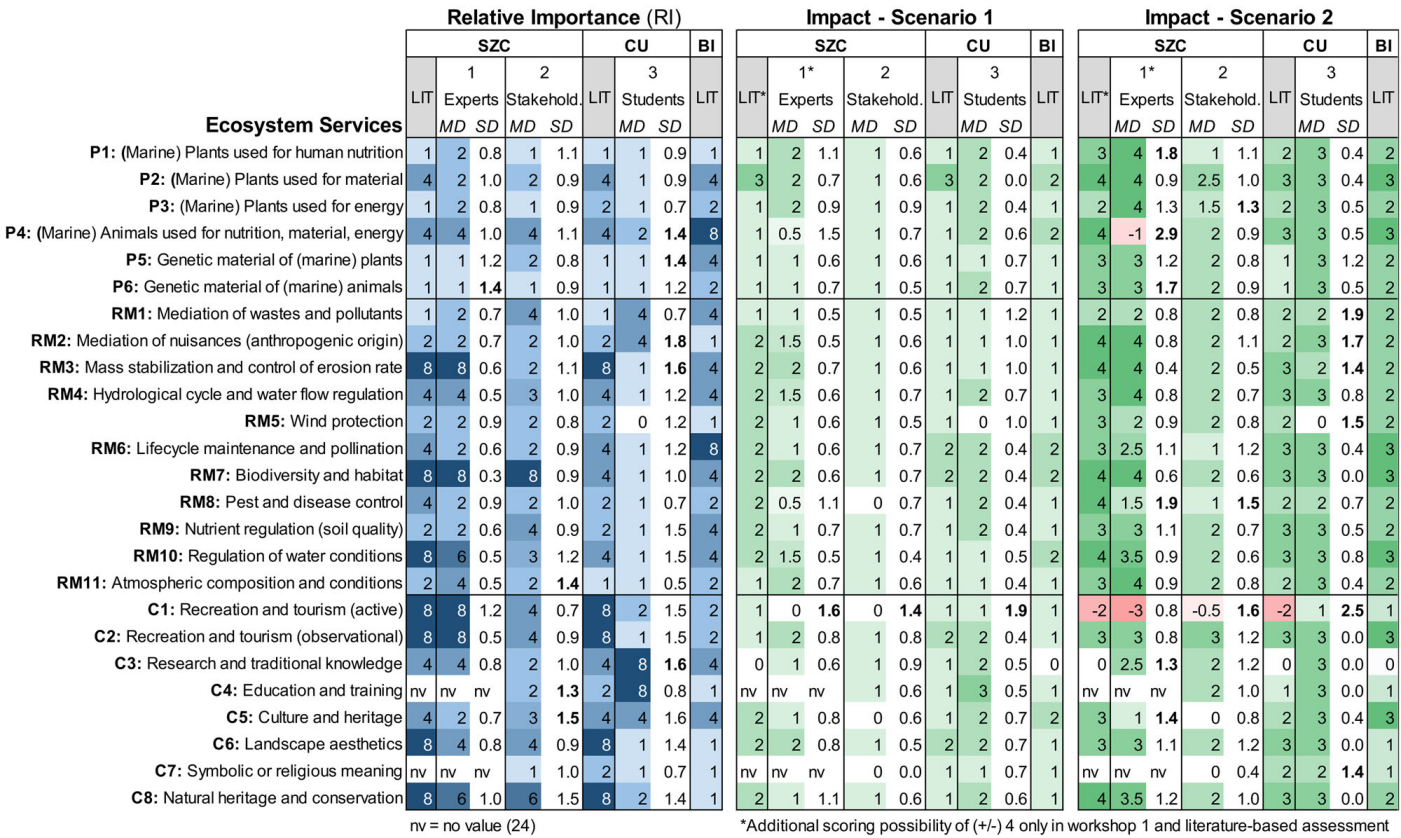
Results of scenario assessments for three lagoons (SZC Szczecin, CU Curonian, BI Bizerte) on their provision of ecosystem services (P Provisioning, RM Regulating and maintenance, C Cultural). The relative importance ranges from 0 (not important) to 8 (very high). The impact score 4 indicates a strong increase in ES provision and −4 a high decrease. Literature results (LIT) are compared to median values (MD) and standard deviations (SD) of three workshops: (1) coastal-management experts, (2) coastal-management stakeholders and (3) student group

With the aim to evaluate the suitability of selected services, we assessed the RI of each service as it is perceived by stakeholders and as reflected in the literature. According to the literature sources, the most important services provided by macrophytes in the Baltic lagoons are among the cultural services, as recreational activities (C1, C2), landscape aesthetics (C6) and nature conservation (C8), accompanied by the service of coastal protection (RM3) (Fig. 5). Experts (Workshop 1) show a rather high agreement with the literature data, which indicates the common pool of knowledge.

For the Szczecin lagoon, the most important services (> 6) according to both stakeholders (Workshop 2) and experts (Workshop 1) are biodiversity and habitat (RM7) and recreation and tourism both active (C1) and observational (C2). However, local stakeholders could provide contrasting information on the RI of some ES. In contrast to expert opinion and literature survey, stakeholder groups downgraded the RI value of coastal protection (RM3), regulation of water quality (RM10), and landscape aesthetics (C6) provided by macrophytes. For the Curonian lagoon (Workshop 3), the most important services (=8) according to the student group are research and traditional knowledge (C3) and education and training (C4). For both lagoons, the most important among the three ES categories are the cultural services with more than 40% (Curonian lagoon) and 60% (Szczecin lagoon) of all services assessed as highly or very highly important (> 4). Regulating and maintenance services are of high importance in the Szczecin lagoon (~4) and only of moderate importance in the Curonian lagoon (~2). Provisioning services show moderate importance in the Szczecin lagoon (~2) and low importance in the Curonian lagoon (~1).

Differences in expert/ stakeholder valuations are reflected within individual scores and comments during discussions (see Online Resource 3). To test data quality and reliability, we use discussion content and standard deviations (SD) as indicators. SD values indicate high agreement among experts (SD = 0.8) and lower agreement among students (SD = 1.2). On the service level, most experts disagreed (SD = 1.3) on the importance of natural heritage and conservation (C8), and culture and heritage (C5), while they mostly agreed (SD = 0.7) on the very high importance of biodiversity and habitat (RM7).

During the discussions, experts argued that, for example, the service biodiversity and habitat (RM7) serves a basis for the whole ecosystem functioning, and is thus pivotal for other services. Some stakeholders assumed that the importance of services changes depending on the actual ecosystem state. One example of this is the service coastal protection (RM3). Its importance increases with higher exposition and hydraulic loads on the coast. Students and some experts stated that cultural services are the easiest to assess, but the most difficult to interpret as they reflect personal preferences and behaviors. Stakeholders considered management implications within their assessment scores. For example, while some only assessed potentially available biomass, others considered if biomass should actually be harvested. Some argued that the potential should not be used to maintain the natural quality, and thus they evaluated too conservative and low. Summarizing, differences in workshop results mainly derive from different interpretations (e.g., needed provision or potential of ecosystem state), subjectivity (especially cultural services) and possible management implications (e.g., impact of harvesting).

Discussions revealed the importance of the participants´ level of expertise with regard to data quality. Stakeholders indicated certain difficulties when carrying out the assessment due to the lack of own expertise, but also due to missing background information. They sometimes felt insecure about their given values. For example, in Workshop 3 students stated their lack of knowledge with regard to current use of marine plants (P2, P3), e.g., local management and further use of reed. Their insecurity is well reflected by their values that differed considerably compared to literature results. Stakeholders (Workshop 2) indicated that they learnt about and became aware of some services, e.g., wind protection of reed belts. Thus, the level of expertise is important in terms of data quality, but stakeholders, usually of lower expertise, experience an increasing understanding of the impact of management measures (here of achieving a GES), leading to an increased acceptance and learning process.

These discussion results are also reflected in Workshop 1, where experts were allowed to change values directly after discussion (in case of misunderstandings), which was done for 3.4% of all values (*n* = 23). Besides, 50% of the experts repeated the assessment after one week. Of all values given, 46% of the high expertise group and 68% of the low expertise group were changed. The results of the high expertise group show a lower standard deviation (SD = 0.89) than the low expertise group (SD = 1.24). Most values of both groups were changed for Scenario 2 (52 and 93%), especially for provisioning (67 and 94%) and cultural services (50 and 100%). The results show that expertise has a positive impact on data quality, but can be improved by increasing the number of participants. Besides, results of the second assessment indicate higher agreement among participants, assuming this being the results of workshop discussions. Due to subjectivity (especially for cultural services), different perspectives and interpretations (e.g., spatial scale), possible tradeoffs and synergies (e.g., motor entanglement), the results show, despite minor differences, that discussions are vital for clarifying misunderstandings and interpreting the scores.

Comparing workshop and literature results of the Szczecin lagoon, we observed main differences for regulating services, where literature results indicate only low importance for mediation of wastes and pollutants (RM1), but very high importance for regulation of water conditions (RM10). In contrast, workshop and literature results of the Curonian lagoon show clear differences, as more than 50% of the services differ more than one scale class. We can state that the student group (Workshop 3) assessed the RI of many services much lower, which can be explained by their lower expertise, misinterpretation and/or misunderstanding. As we find only minor differences between expert and stakeholder results (except from students) compared to literature data, we assume a high compatibility of expert, stakeholder and literature-based assessments in terms of data reliability.

Our literature results for the Bizerte lagoon differ considerably compared to the results of the Baltic lagoons. The main differences are found among cultural services, where the average importance in the Bizerte lagoon is lower (RI ~ 2) than in the Baltic lagoons (RI ~ 6). Provisioning services are more important in the Bizerte lagoon (RI ~ 4) than in the Baltic lagoons (RI ~ 2). Regulating and maintenance services were assessed similarly of moderate importance (RI ~ 3). While for the Baltic lagoons, cultural services are perceived most important according to our results, these are almost negligible for the Bizerte lagoon (Mediterranean Sea), where provisioning services are currently most important.

Reasons for these differing results of the Baltic and Mediterranean lagoons can be multifold, but we assume that our results mainly reflect the different socio-cultural and economic conditions, as well as species representing macrophyte communities. By this, we identified the most important services for each lagoon, which are of highest interest for the regional management and policy makers. From this, we learnt that the developed list of services is suitable and applicable in an international context, as all selected services are assessed at least of low relative importance for all lagoons.

### Assessment of Management Scenarios Based on ES Provision

The aim of the scenario assessment is to test the applicability of our approach for management purposes, specifically here to assess the impact of achieving a GES of lagoons, using expert, stakeholder and literature data. Our results indicate the perceived impact of scenarios on ES provision (Fig. 5). For the Szczecin and Curonian lagoon, 24 of 25 services provided by macrophytes show low to moderate positive impacts (increase in ES provision) for Scenario 1 and a moderate to strong positive impact for Scenario 2. In Scenario 1, results indicate the strongest increase (+3) for marine plants used for material (P2). While moderate macrophyte coverage (in Scenario 1) shows a slight positive impact on active recreation and tourism (C1), in Scenario 2 this turned into a clear negative impact by strong macrophyte coverage.

In Scenario 2, results for Szczecin lagoon show one negative outlier for marine animals used for nutrition, material or energy (P4), also showing the highest disagreement among stakeholders in this regard (SD = 2.9), probably reflecting difficulties to forecast fishery landings along with increasing macrophyte coverage. In general, the experts and student group assessed a stronger positive impact of the higher macrophyte coverage on all services (similar to literature data) than the stakeholder group which assessed more conservatively. However, the workshop and literature results of both Baltic lagoons show only minor differences. While our results generally indicate a positive impact on ES provision by improving the lagoon´s ecological state, the strong macrophyte coverage (in Scenario 2) marks a clear tipping point, as experts and stakeholders perceived a strong negative impact on active recreation and tourism (C1).

Despite minor differences of workshop results, during discussions experts showed opposing arguments based on high subjectivity and personal preferences. For example, while one expert finds that reed belts block the view of the water reducing recreational quality (C1), others perceive reed as a habitat and enjoy it for observational purposes (e.g., bird watching). Experts discussed the impact on carbon sequestration (RM11), which highly depends on the perspective (small-scale or large-scale) and interpretation of the impact (e.g., local or global). Stakeholders mentioned tradeoffs and synergies between services. For example, they emphasize on the conflict between regulating and cultural services, i.e., macrophytes providing clear and clean water for bathing versus entanglement of macrophytes in sport boats. Besides, it was pointed out that emerged macrophyte stands are appealing to tourists, as they also use reed stands to hide and search for wind protection, but also often limit the access to the water. Moreover, stakeholders discussed the role of positionality and personal evaluation behavior, thus people´s “habit” to assess more extreme, conservative and moderate or optimistic and pessimistic. Thus, our approach is useful to hear the concerns and questions raised by society and in turn use this information for revising the elements of scenarios e.g., include macrophyte management options such as reed cut.

In the Bizerte lagoon (literature data only) we find only minor differences from the Baltic lagoons derived during workshops and literature-based results. The main difference in Scenario 2 is that for the Bizerte lagoon we also find a slightly positive impact of increasing macrophyte coverage on recreation and tourism (active) (C1). This can be explained by different recreational uses of the Baltic and Mediterranean lagoons, as in the Bizerte lagoon water transparency plays a more important role due to diving activities, whereas increase in macrophyte coverage would imply improvement of diving sites. For landscape aesthetics (C6) we only find a low increase, which is explained by their function as buffer zone and trap of (plastic) pollution, i.e., extended macrophyte stand would also accumulate more litter and therefore reduce the overall aesthetic value provision. In Mediterranean lagoons, fisheries and aquaculture play a significant role, thus the economic value of ecosystem outputs (e.g., fish, shellfish) is very important (El Mahrad et al. 2020). Besides, there is higher potential for economic income sources within blue economy sectors, for example seaweed cultivation and salt production, as biomass is often abundant but not exploited (El Mahrad et al. 2020; Ktari et al. 2022). Seaweed cultivation bears especially in Tunisia and North Africa great potential as a mitigation measure to preserve the intensively used coasts by industrial, urban and touristic activities (Ktari et al. 2022). In general, for the impact scenarios there are only minor differences between lagoons, ES, service categories and standard deviations (i.e., stakeholder agreement). Therefore, we can conclude that our scenario assessments, both workshop and literature-based, are suitable and applicable for comparative studies on lagoons of different ecological states, geographical locations (Baltic vs. Mediterranean Sea) and socio-cultural contexts (e.g., different recreational activities).

### Spatial Extrapolation - Implications for Management

**Fig. 6:**
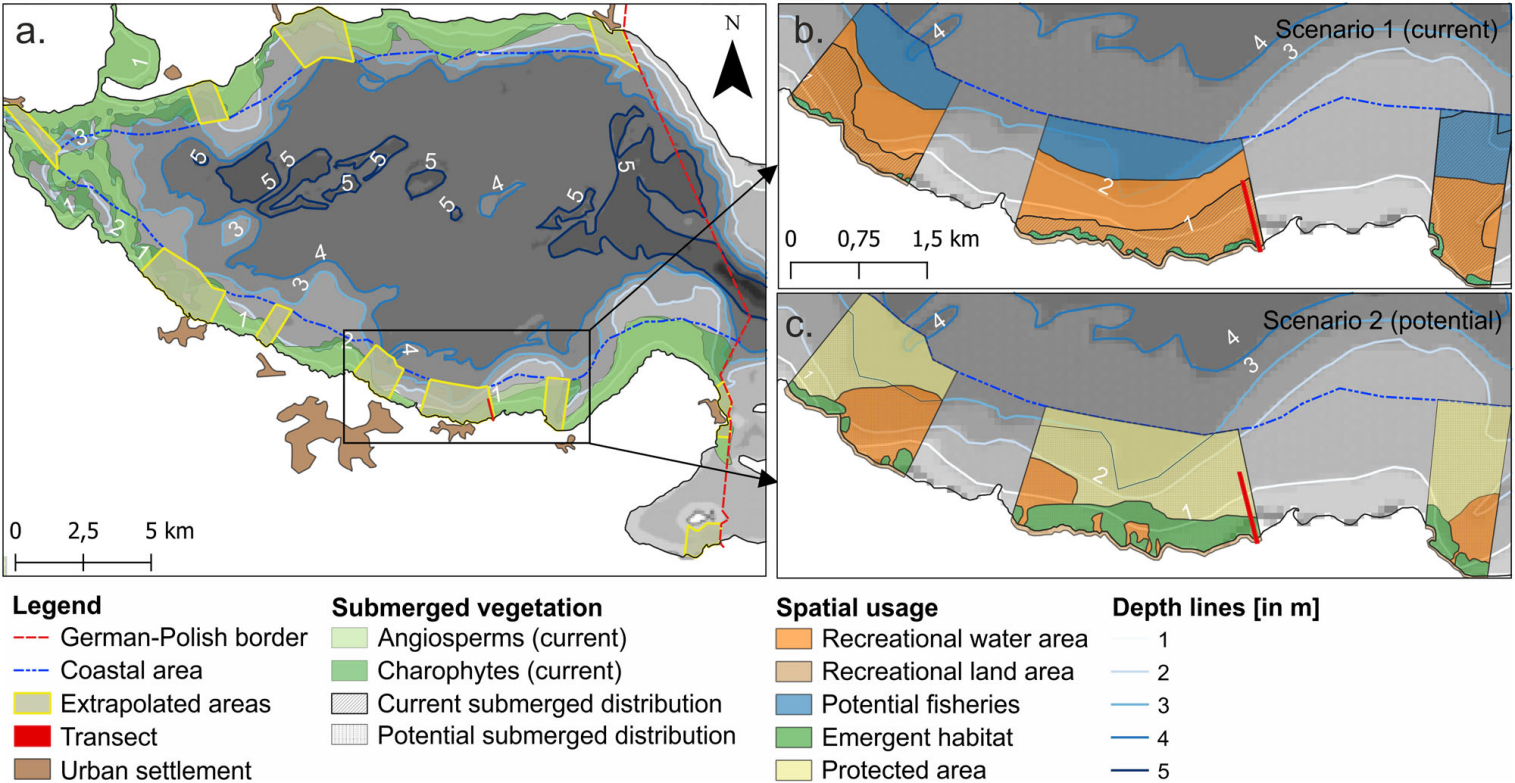
Spatial extrapolation maps of the Szczecin lagoon showing all extrapolated areas of the German part Kleines Haff (**a**), the current spatial use mainly recreation on water and fisheries (**b** Scenario 1) and the potential spatial use under nature protection and GES (**c** Scenario 2)

In order to transfer our small-scale results of the chosen transect to large-scale system level, we extrapolated scenario results exemplarily to designated areas of the Kleines Haff (German part of the Szczecin lagoon). Our scenario transect (see Fig. 3) has an area of approximately three hectares (ha). Current submerged vegetation of the Kleines Haff covers an area of 5795 ha (Fig. 6a). Potential submerged vegetation (including angiosperms and charophytes) may increase by 78% to an area of 10,334 ha under the premise of achieving a GES and a growth limit of up to 3 m (according to Porsche et al. 2008). For extrapolating scenario results to the whole area of the Kleines Haff, we identified a possible area of 2137 ha or 25% of the coastal zone. Exemplarily, we focused on three extrapolated areas surrounding our scenario transect (Bellin beach). In Scenario 1 under current use or state, emergent vegetation (mainly reed belts) covers only small areas of 20 ha (3% of the total extrapolated area) (Fig. 6b), while under potential use or nature protection in Scenario 2 it increases to 17% (of the total extrapolated area) or 109 ha (Fig. 6c). Due to discussions during scenario assessments, we further subdivided recreational use (or area) for the extrapolation into activities on water and on land (mainly beach area). While water area for recreational use decreased from 397.57 ha (61%) to 131.13 ha (20%), recreational use on land did not change in area. Extrapolation results indicate a strong spatial tradeoff and conflict between recreational use (here mainly on water) and expansion of macrophytes (i.e., submerged and emergent vegetation).

Compared to the impact values of our scenario results (Fig. 5), the main difference is that the decrease in beach activities is not visible in our extrapolation results, as the spatial area for recreational use on land is not subject to changes in macrophyte distribution. However, from our scenario results we learnt that the increase in emergent and submerged vegetation is perceived as a cause for reduced recreational activities also and especially on land. For example, bathing opportunities are limited by reed belts prohibiting water access, or beaches just lose their attractiveness due to the blocked view. Therefore, macrophyte recovery by achieving a GES cannot be a desired state over the whole areas, as it is significantly inhibiting coastal tourism being an important economic driver of the region. In such case some macrophyte removal from the designated areas will be necessary.

By identifying areas for extrapolation, we also identified areas that are of high interest and importance for management and policy measures. Extrapolated areas are highly important for the tourism sector. Thus, extrapolation results can serve as a basis for decision-making when designating, for example, nature protected areas, fishing grounds, water sport areas or other use rights. In summary, while some human activities (or spatial usages) show clear tradeoffs in terms of space (e.g., vegetation vs. recreational area on water), other spatial usages even provide synergies (e.g., vegetation and nature protection) or do not compete for space at all (e.g., fisheries and recreational activities on water).

We learnt that scenario results can be extrapolated to entire water body level when areas share similar characteristics as the transect. Extrapolation results even combine the importance of services (or spatial uses) with possible macrophyte expansion. However, the scenario and extrapolation results represent a very broad macrophyte composition and coverage, often considering only monocultures of reed belts. Therefore, our extrapolation results are suitable to discuss possible management outcomes for human activities. However, more scientific research is needed to estimate the total scenario impact on regulating and maintenance services.

### Assessment of Different Macrophyte Habitats based on ES Potential

**Fig. 7:**
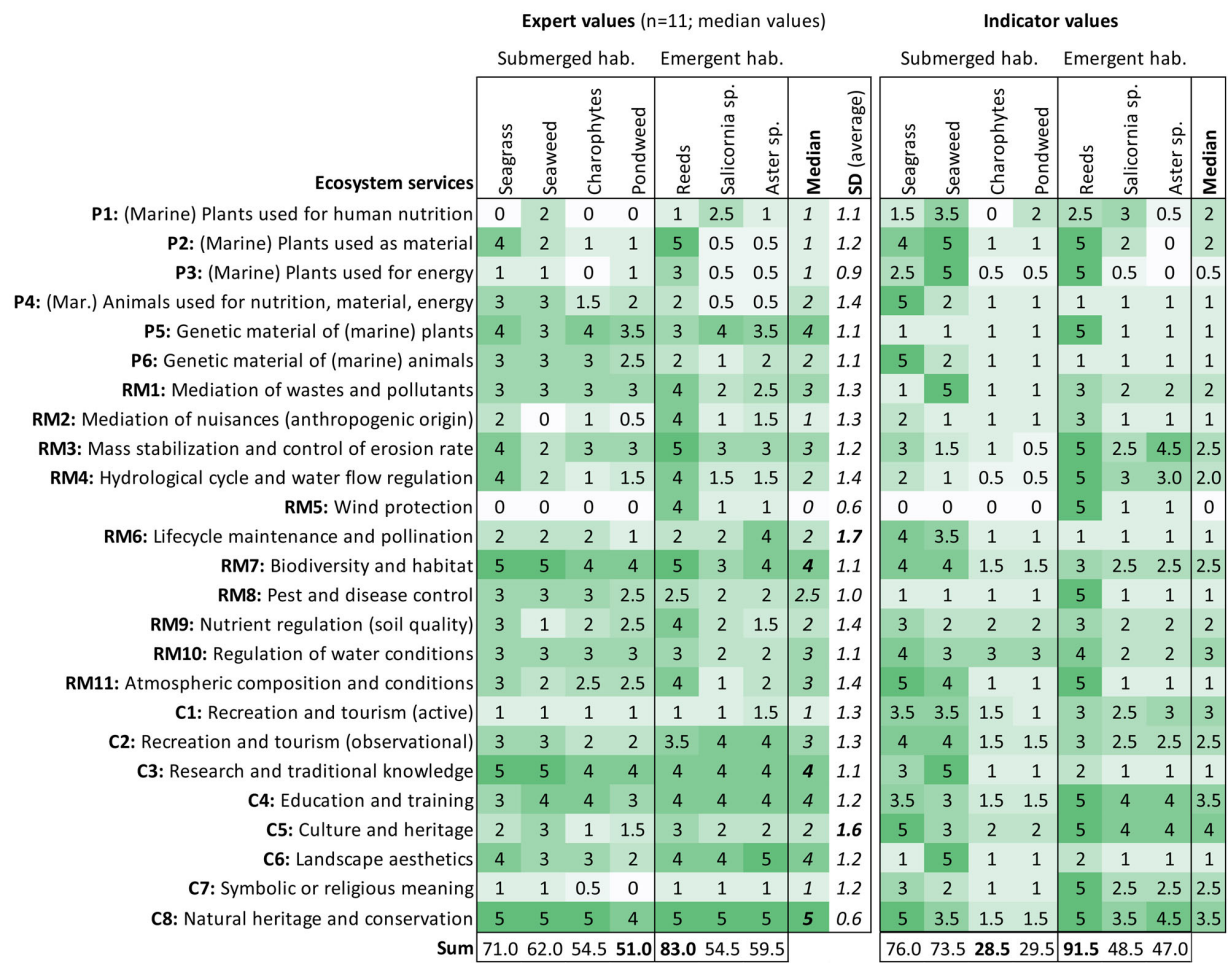
Results of ES assessment (P Provisioning, RM Regulating and maintenance, C Cultural) in macrophyte habitats of the Baltic Sea. Values indicate no potential (0) to very high potential (5). Median values and standard deviations (SD) from macrophyte experts and literature-based results are shown for each habitat (listed according to the sea-land gradient)

In order to test the applicability of our approach for broader coastal environmental conditions and macrophyte diversity, we assessed the ES potential of macrophyte habitats, differentiating between submerged (i.e., seagrass, seaweed, charophytes, pondweed) and emergent macrophyte habitats (i.e., reeds, salt meadows dominated by *Salicornia spp*. and by *Aster spp*.). Shown in Fig. 7, the experts assessed the highest overall potential (calculated by total sum of all ES scores for each habitat) to be provided by reeds and tall forb communities (sum of scores: 83 out of a maximum of 125), followed by seagrass beds (71) and seaweed communities (62). The lowest overall potential is indicated for pondweeds (51). Our literature-based results show in general a similar trend as the expert, showing main differences for the habitats of charophytes (29.5) and pondweed (28.5) that show the lowest potential. For detailed literature-based results see Online Resource 5.

Based on expert results, the individual services with the highest potential (indicated by the median value of each ES) provided by macrophytes are natural heritage and conservation (median value of C8: 5), education and training (C4: 4), research and traditional knowledge (C3: 4), genetic material of (marine) plants (P5: 4) and biodiversity and habitat (RM7: 4). The results depict a slight land-sea gradient, with decreasing potential from sea (submerged) to land-dominated macrophytes, and from marine to brackish and freshwater habitats. For example, seagrass beds and seaweeds show very high potential for biodiversity and habitat (RM7: 5), while salt meadow species show only moderate to high potential (< 4). Contrarily, emergent habitats show higher potential for “visual” services like landscape aesthetics (C6: > 4), but also for observational recreation (C2: > 3.5). In general, expert results of all habitats show the highest ES potential for cultural services (median: 3), followed by regulating and maintenance services (median: 2.5), and lowest ES potential for provisioning service (median: 2). Similarly, literature-based results indicate the same trend with highest ES potential for cultural (median: 2.5), then regulating and maintenance (median: 2) and provisioning service (median: 1).

As an indicator for data quality, we focused on standard deviations (i.e., expert agreement). Most disagreement among experts (high SD), in general for all habitat, can be seen for lifecycle maintenance and pollination (RM6: 1.7) and culture and heritage (C5: 1.6) (for result of individual experts, see Online Resource 4). Due to the high expertise among selected experts, the data quality is high, as SDs are on average relatively low (~1.2), with the highest for seaweed communities (~1.3) and the lowest for charophytes (~1.1).

We carried out a correlation analysis to identify the relevance of expertise for the quality of results and to identify possible tradeoffs and synergies between the services. Testing the dependence of ES potential results on the level of expertise, we find moderate correlation (Spearman’s rank correlation coefficient’s *r* = 0.5 to 0.79) for 12.6% of all services and strong correlation for 3.4% (r > 0.8) of all services, especially provided by salt marshes dominated by S*alicornia* (24%), charophyte habitats (20%) and reed and tall forb communities (20%). Regarding the dependence between ES potential results, the data show strong positive correlations between biodiversity (RM7) and research (C3) when provided by seaweed (0.88), seagrass (0.95), and pondweed habitats (0.91). Analysis results show a slight negative correlation trend between provisioning services and regulating as well as cultural services, and partly even among provisioning services themselves. Contrarily, regulating services show a slight positive correlation to cultural services and between regulating services themselves. Summarizing, the low correlation of the level of expertise and of services can be neglected, while the correlations between services and ES categories may indicate possible tradeoffs mainly between provisioning services and others.

By comparing the ES potential of macrophyte habitats to the RI values of the scenario assessments (Fig. 5), we can identify which habitat has highest potential (5) for contributing to the most important services (RI = 8) provided by Baltic lagoons. Thereby we assume that this relates to the actual ES provision. While reed shows the highest overall potential among lagoon macrophyte habitats, it probably contributes mainly to coastal erosion prevention (RM3). Macrophyte habitats that are main providers for biodiversity (RM7) are reeds, seagrass beds and seaweed communities. The most striking service we found is active recreation (C1), as it is of very high importance for Baltic lagoons, but all habitats show only low or very low ES potential (< 1.5). Despite this, results of the scenario assessments even showed a strong negative impact on active recreation (C1) by macrophyte expansion perceived by stakeholders. Contrarily, for observational recreation (C2), results show clearly the highest ES potential (> 3.5) of emergent macrophyte habitats (i.e., reeds, salt meadows dominated by *Salicornia spp*. or *Aster spp*.). Similarly, the emergent habitats, especially salt meadows dominated by *Aster spp*., also contribute mostly to landscape aesthetics (C6). Regarding natural heritage and conservation (C8), all habitats bear high to very high potential.

## Discussion

### Methodological Assessment

Our comprehensive list of services and assessment indicators is based on a common international classification (i.e., CICES) and literature, our developed management scenarios and macrophyte habitats build upon definitions of the WFD, HD, and EUNIS, both being beneficial for general applicability and transferability of approaches to other Southern Baltic Sea and Southern Mediterranean lagoons. Our approaches offer several opportunities as well as face some methodological limitations that we want to point out and that need to be addressed in future studies.

The suitability of the selected ES list was confirmed by stakeholders, assessing all listed services to be at least of low importance. Our ES list allows for integration of both scenario and habitat approaches, and allows to develop the scenarios with different dominant macrophyte species in the specific local growth conditions. We recommend minor adaptations of ES descriptions and examples to local and case-specific conditions. For example, while roof thatching is a good example for reed harvesting in the Baltic Sea, this does not apply in the Mediterranean Sea.

Due to a high complexity or the lack of harmonized indicator schemes within monitoring and assessment of ES (Czúcz et al. 2018), especially for coastal and marine services (von Thenen et al. 2020), our indicator list can serve as a solid base for assessing ES provided by macrophytes. The number of pre-selected indicators (174) constitutes the limit for such selection process due to experts´ time constraints. For each service three to four indicators were chosen and ranked by experts. In case of low data availability, indicators can serve as additional guidance and description for stakeholders and experts in order to improve data quality (i.e., common understanding). However, we learnt that some of the listed indicators were too general and could not be used to differentiate between single macrophyte habitats, e.g., contribution to coastal tourism (income € per year). Due to a lack of data and when assessing on large-scale habitat level indicators were partly difficult to apply. However, we tried to use at least one indicator from the developed list complemented by “Number of Web of Science (WoS) articles” indicating the relevance of given keywords, which we assume represents the ES potential. Despite this, we assume that indicators work well on specific water body level, e.g., for well-defined and precise study areas, where data availability is higher.

Scenario methodology is widely applied in developing spatial planning with integrated ecosystem services assessment or modelling more in the terrestrial (e.g., Kabaya et al. 2019). than marine areas (e.g., Farella et al. 2020). Scenario assessment appeared to be a successful tool to start discussions among participants supporting decision-making processes, e.g., for marine mussel cultivation (Ritzenhofen et al. 2022). As shown by Schernewski et al. (2019), this approach can be applied to assess the implementation of EU policies, for example, the measure of WFD to achieve a GES. For scenario assessments, only low expertise of participants is needed allowing for broad stakeholder involvement. The approach focuses on perceptions, identifying misunderstanding and finding a common understanding (Robbe et al. 2021), as also stated by stakeholders of this study. Another opportunity for application, as we learnt during the discussion, can be as a learning tool for awareness raising and for teaching graduate students (i.e., lecture, thesis), which is also supported by Rodríguez-Loinaz and Palacios-Agundez (2022) and Barracosa et al. (2019). Although the level of expertise being important in terms of data quality, a low level of stakeholder expertise may lead to an increased understanding of management measures and thereby to an increased acceptance of such. Regarding the transferability of scenario assessments, this approach can be used in general not only for coastal areas that have similar ecosystem characteristics (i.e., Baltic lagoons), but also for contrasting systems (i.e., Mediterranean lagoon).

Spatial extrapolation represents a common method for assessing and mapping ES (Martínez-Harms and Balvanera 2012; Andrew et al. 2015; Le Clec’h et al. 2018). Our spatial extrapolation approach has the limitation that extrapolated areas and scenarios build upon simplified assumptions (e.g., macrophyte expansion up to 3 m depth) and have a strong focus on touristically important areas. We further differentiated between recreational activities on land (e.g., beach area) and on water (e.g., boating), which stakeholders criticized for being merged in scenario assessments. However, the extrapolation results only show changes by different scenarios in water area for recreational activities, but no change in land area. This neglects changes in recreational activities on land, as scenarios will affect land not in area but in activity type (e.g., no sun bathing because of limited water access for swimming). We learnt that scenario results are suitable to be extrapolated to the entire water body level when areas share similar characteristics as the transect. However, for further studies we recommend to include transects of different focus, e.g., touristic use, fisheries, and nature protection. Despite these limitations, the approach can serve as a basis for decision making, for example, when designating nature protected areas, fishing grounds, water sport areas or other use rights. Especially for spatial planning measures, this approach could support local spatial planning processes (compare Schernewski et al. 2023) by identifying areas of highest interest for different spatial uses (tourism vs. nature protection). For this, further development of the approach is needed, for example, by integrating ES assessment results (i.e., importance of services and impacts of spatial planning measures on services) and concrete spatial land and/or water use data.

Our habitat assessment was tested for the Baltic Sea and considered to be suitable for identifying differences between ES of emerged and submerged macrophyte habitats when assessing comparatively but not individually. Similar approaches exist for terrestrial, coastal and marine ecosystem types in Northern Germany (Müller et al. 2020) and ecosystems across the land-sea interface in the Baltic (Schumacher et al. 2021). We learnt that the application on a Baltic Sea wide level works well when using expert knowledge, but only very limited when using indicators. Regarding the literature-based results, there is a strong bias towards representation in literature, as we used the relevance indicator (i.e., number of WoS articles) for 60% of the services, complementary or single. We can state that there is a discrepancy between expert opinion and literature with regard to the service potential in particular of charophytes and pondweed. Results clearly show the need for expert knowledge when assessing macrophyte habitats on a large-scale due to the lack of literature data for selected habitats (i.e., charophytes and pondweed). For future studies we suggest to apply our habitat assessment to specific lagoons, thus using smaller and well defined spatial areas where data availability is higher and indicators are more easily applicable. This approach allows for comparative assessments of individual macrophyte habitats (e.g., seagrass beds), possibly in different seas. We assume a good transferability of our habitat approach to other systems internationally, for example Mediterranean lagoons, by mainly using expert knowledge where data availability is possibly low.

### Implications for Management and Policy Implementation

As the status of macrophytes is still not in a good or high ecological state for around 50% of all transitional and coastal waters in the EU (EEA 2018), there is a need for supporting policy implementation to achieve its goals (i.e., achieving GES). This study provided holistic approaches of ES assessments, specifically targeting macrophytes, to support coastal management and policy implementation. Transferring main results of this study to current coastal management and policy implementations, we learnt that our approaches can support the evaluation of different management measures. Main areas of macrophyte management in coastal areas that we identified by the high ES potential and importance of macrophytes are 1) nature protection (incl. climate protection), 2) coastal protection, 3) blue economy, and 4) coastal tourism, which are also reflected in relevant EU policies (WFD, HD, Sustainable Blue Economy).

First, the protection of macrophyte habitats is addressed by several EU policies, mainly the WFD and HD. However, measures of implementation are partly unsuccessful as either assessment results indicate only little effect or are not sufficiently represented in the results (BMUB/UBA 2016). For example, benefits of protecting nature are often economically invisible and regarded as intrinsic (TEEB 2010). With our approaches we can justify the values of macrophyte habitats and thereby the benefits of protecting them. For instance, we can demonstrate the value of the halophyte *Aster tripolium*, a red list species, located in the protected area “Smeltes botaninis draustinis” (Klaipeda, Lithuania) that is in the industrial harbor area of the Curonian lagoon (Olšauskas et al. 2013). Additionally, macrophyte habitats play a role within climate change mitigation measures, for example by reed belts (Buczko et al. 2022) and sea grass beds (Stevenson et al. 2022) as carbon storages. Summarizing, our results can support the implementation of management and policy measures by explaining the benefits to humans, for example, of achieving the GES (e.g., restoration of macrophyte habitats by reducing agricultural nutrient loads) and of enhancing the biodiversity of macrophyte habitats (e.g., to protect rare species and prevent monocultures).

Second, especially reed belts (Coleman et al. 2023) and seagrass beds (Chen et al. 2022) are of great importance for coastal protection by reducing wave energy in the foreshore area. A recent project specifically targets planting and reforestation of seagrass beds in the Baltic Sea (SeaStore). In the Curonian lagoon, reed belts were already used as dune protection in the 1960s when planted in front of Juodkrante in order to decrease coastal erosion (Galiniene et al. 2019). This example reflects well on our ES results showing the tradeoff between coastal protection (i.e., planting reed belts), biodiversity (i.e., loss by monoculture) and tourism (i.e., blocking view or limiting access), but also one synergy by providing material for further use when harvesting reed regularly. In the context of sea-level rise and storm surges, the importance of emergent macrophytes (i.e., reed), in particular, may increase even further due to higher demand of coastal protection.

Third, the EU Sustainable Blue Economy Strategy (EC 2020a) recognizes the economic potential of marine macrophytes and their biomass, also reflected in our ES potential results for provisioning services (e.g., plant biomass as material for further processing). Besides, Lillebø et al. (2017) highlight the potential of marine macrophytes in the blue energy sector by substituting non-renewable energy sources, for example, biogas production or direct combustion (Wichmann 2017). Within the EU Farm-to-Fork strategy, which includes specifically aquaculture guidelines (Council of EU 2020a; 2020b), macrophytes can be farmed and harvested for the purpose of human nutrition (Wells et al. 2017), either by saline agriculture (Nikalje et al. 2018) or by marine aquaculture. The latter, commercial seaweed farming in the Baltic Sea bears not only economic potential, but also reduces nutrient loads to combat eutrophication (Kotta et al. 2022). Besides, the genetic material of macrophytes bears the potential for pharmaceuticals and cosmetics (Puchkova et al. 2022), as well as for further processing and use of the material, e.g., thatching (Karstens et al. 2019). Concluding, the potential of macrophyte habitats to deliver provisioning services for blue economy (e.g., food, feed, fuel) is given, but often limited due to poor or uncertain economic viability, requiring synergies (i.e., seaweed farming for biomass production and for nutrient removal).

Forth, with regard to coastal tourism, macrophyte management is pivotal. For example, at sport boat harbors, macrophytes are either removed or destroyed by frequent boating activities or contrarily cause damage to motors by entanglement in macrophytes (Verhofstad and Bakker 2019). Both results in a need for management measures, i.e., cutting or removing macrophytes, which can also cause high costs for municipalities if not further used economically (Wichmann et al. 2017). As shown in our scenario results of cultural services (high macrophyte coverage), high-growing reed belts can be also seen as nuisances to tourists by blocking the view, or contrarily being pivotal for the aesthetic experience (Karstens et al. 2019). Though results show in general a positive impact of macrophytes and their recovery (e.g., within achieving the GES), the question remains, if the GES in terms of macrophytes is always a desired state? Due to spatial tradeoffs and conflict between recreational use and expansion of macrophytes, management and policy measures need to be clearly adapted to the regional importance of human activities (i.e., demand for ES). Macrophyte expansion within the GES may significantly inhibit coastal tourism, which can be an important economic driver of coastal regions. Our approaches can be used to identify areas of high importance for tourism or, for example, biodiversity hotspots. Therefore, management and policy measures could target specific areas of high or low importance of ES provision (or demand) to avoid tradeoffs and to use synergies.

## Conclusion

To the best of our knowledge, this is the first systematic ES assessment of macrophyte habitats comparing their ES provision and potential under different management scenarios (i.e., different ecological states) and in different seas (i.e., Baltic and Mediterranean Sea).

Macrophytes are beneficial to humans. However, management measures have to be in accordance with the spatial use of coastal areas. Our approaches give fast and easy results on the perception of management measures, here macrophyte expansion and improvement of ecological states. This research has shown that macrophytes are generally perceived as beneficial to humans. Nonetheless, macrophytes are also considered to mainly inhibit coastal tourism and recreation, which are the most important services in the Baltic lagoons. This finding strengthens the need to integrate spatial use data and ES assessment results for identifying specific tradeoffs and synergies of management measures. As the understanding of the good ecological status as dominance of macrophyte habitats within the WFD is often too narrow and bound to main benefits, i.e., water transparency, our holistic ES assessment approaches might be beneficial for multi-sectorial management. We applied and integrated different assessment approaches (i.e., scenario and habitat level) as well as different data sources, namely socio-economic data (by stakeholder and experts) and biophysical data (i.e., indicator-based). It is unfortunate that the indicator-based assessments are highly limited due to the lack of data on the selected habitats. Inspite of its limitations, this study presents two ES assessment approaches that are internationally valid and applicable for assessing macrophyte habitats and coastal lagoons. We further recommend to carry out future research applying these approaches to other coastal habitats worldwide, e.g., mangroves, ice-dominated habitats (other climate zones, tropical, ice).

### Supplementary information

Online Resource 1

Online Resource 2

Online Resource 3

Online Resource 4

Online Resource 5
