# Supplementary material for: Assessment of Ecosystem Services Provided by Macrophytes in Southern Baltic and Southern Mediterranean Coastal Lagoons
Source: Environ Manage. 2024 Mar 7;74(2):206–29. doi: 10.1007/s00267-024-01955-9 (PMC11227447; doi:10.1007/s00267-024-01955-9)
Supplement: Supplementary file 1 — Online Resource 1 [file 267_2024_1955_MOESM1_ESM.docx]

**Detailed descriptions on macrophyte habitats for assessing ES potential**

based on definitions and categories from EU Habitats Directive (Natura 2000) and EUNIS (2022)

**Seagrass beds on mixed sediments habitats***^[[1]](#footnote-1)^***:** Submerged rooted plants cover at least 10% of the seabed (mud or sand), from which the common seagrass species account for more than 50% of the biovolume. Salinity ranges from low to moderate. Exposure ranges from low to moderate. Depth ranges in photic zone from about 0.1 to 4 meters. Characteristic species are *Zostera marina* and *Zostera noltii*.

- Classification by Natura 2000: Open sea and tidal areas; Zostera beds (1150, 1130, 1160)
- Classification by EUNIS: Baltic hydrolittoral mixed sediment habitats characterized by *Zostera marina* (MA3326, MA4325), Baltic infralittoral mixed sediment dominated by *Zostera marina* (MB4325)

**Macroalgae communities on mixed sediment habitats***^[[2]](#footnote-2)^:* Coverage of perennial and/or annual attached algae is at least 10%, and more than other perennial/annual attached erect groups. Salinity ranges from low to moderate. Exposure ranges from moderate to high. Depths range in photic zone down from about 0.5 meters to 5 meters and deeper in clear waters. Characteristic species are *Fucus spp*., *Furcellaria* *lumbricalis*, *Deleseria* *sanguinea*, *Cladophora* *spp*. and *Ulva* *spp*.

- Classification by Natura 2000: Open sea and tidal areas; Benthic algal communities (1150, 1130, 1160)
- Classification by EUNIS: Baltic infralittoral mixed sediment characterized by annual algae (MB43D)

**Charophytes on mixed sediment habitats*:** Submerged rooted plants cover least 10 % of the seabed and more than other perennial attached erect groups. Out of the submerged rooted plants, Charales constitute at least 50 % of the biovolume. Salinity range is below 6 psu. Exposure range is low. Depth ranges in photic zone from about 0.2 to 7 meters. Characteristic species are *Chara* *aspera*, *Chara* *tomentosa*, *Tolypella* *nidifica*, *Chara* *horrida*, *Chara* *baltica*

- Classification by Natura 2000: Open sea and tidal areas; Submerged angiosperms (1150, 1130, 1160)
- Classification by EUNIS: Baltic hydrolittoral mixed sediment characterized by Charales (MA4323)

**Pondweed on mixed sediment habitats:** Submerged rooted plants cover least 10 % of the seabed and more than other perennial attached erect groups. Out of the submerged rooted plants, pondweed constitutes at least 50 % of the biovolume. Salinity range is below 6 psu. Exposure range is sheltered. Depth ranges in photic zone from about 0.2 to 4 meters. Characteristic species are *Potamogeton perfoliatus*, *Stuckenia* *pectinata* and *Potamogeton rutilus*.

- Classification by Natura 2000: Open sea and tidal areas; Emergent angiosperms (1150, 1130, 1160)
- Classification by EUNIS: Baltic hydrolittoral coarse sediment characterized by *Potamogeton perfoliatu*s and/or *Stuckenia pectinatas* (MA3321)

**Reeds and tall forb communities:** Baltic bottoms in the photic zone with at least 90 % coverage of sand. Emergent vegetation covers least 10 % of the seabed and more than other perennial attached erect groups. Out of the emergent vegetation, reed species constitute at least 50 % of the biovolume. Salinity range covers all gradients. Exposure range is moderate. Depth range is approximately below 3 meters. Characteristic species: *Phragmites* *australis*, *Juncus* *spp*. and *Carex* *spp*.

- Classification by Natura 2000: Open sea and tidal areas; Reeds and tall forb communities (1150, 1130, 1160)
- Classification by EUNIS: Baltic hydrolittoral littoral coarse sediment dominated by common reed (MA3311)

**Saltmarshes dominated by *Salicornia* and other annuals**: Formations composed mostly or predominantly of annuals, in particular Chenopodiaceae of the genus Salicornia or grasses, colonizing periodically inundated muds and sands of marine or interior salt marshes. Characteristic species are *Salicornia* *europea* and *Suaeda* *maritima*.

- Classification by Natura 2000: Atlantic and continental salt marshes and salt meadows; Salicornia and other annuals colonising mud and sand (1310)
- Classification by EUNIS: *Salicornia* spp. pioneer saltmarshes (MA2252)

**Salt meadows dominated by *Aster tripolium***: Communities of the lower part of the coastal saltmarshes of the Atlantic and its connected seas co-dominated by *Puccinellia* *maritima* and *Aster* *tripolium*.

- Classification by Natura 2000: Atlantic and continental salt marshes and salt meadows; Atlantic salt meadows (1330)
- Classification by EUNIS: *Aster tripolium* var.discoides pioneer saltmarshes (MA2259)

1. Mixed sediments habitats are defined as Baltic bottoms in the photic zone with more than 10 %, but less than 90 % coverage of both hard and soft substrata. [↑](#footnote-ref-1)
2. Ibid. [↑](#footnote-ref-2)
